# Dental Commencements

**Published:** 1889-05

**Authors:** 


### Editorial.

### DENTAL COMMENCEMENTS.

Another college year has ended, and a large number of dental graduates have been cast upon their own resources, some to succeed, some to fail. Many will find that the time spent has been too short fully to prepare them for the manifold duties, which will only too rapidly devolve upon them; and in their perplexity they will wish that they had spent another year, aye, two, at their alma mater. The question is often asked in the East, where do all the dental graduates go? When the boundless possibilities of the West, with its rapidly growing population are considered, it does not take a wiseacre to say *West.* But not all go West; many remain at home to take the places of their preceptors, who on account of age or failing health, are intending to retire from practice, and the young graduate by reason of his better qualifications finds little difficulty in holding the practice. When it is taken into consideration that the estimated number of dentists in practice who hold degrees is not more than ten or fifteen per cent. of the entire body, it is easy to see that it will be many years before there will be a genuine glut in the profession, and when really good men, fully qualified for practice, will experience trouble in finding a place where they can earn a good living; especially as the standard for graduation is being raised, and the doors barred against the entrance of illiterate and ill-prepared men into the profession. Let the good work go on. There is always room at the top, and we welcome the boys into the ranks.

We acknowledge the receipt of programmes and lists of graduates from the following institutions:

The Third Annual Commencement of the American College of Dental Surgery was held on Monday, March 25, 1889, in Chicago, Ill. The doctorate address was by C. T. Hood, A. M., M. D., and the valedictory by J. A. Whipple, D. D. S. Degrees were conferred by the President, L. D. McIntosh, M. D., D. D. S., upon twenty candidates, out of a class numbering —— matriculates. The senior class represented five different states. Increase of matriculates over 1888 ——. Per cent. of graduates to matriculates for 1889, ——, for 1888, 18; average for two years, ——.

The Baltimore College of Dental Surgery held its 49th Annual Commencement in the Lyceum Theatre, March 15, 1889. The music was by Prof. Adam Itzell's orchestra, and we notice the innovation of dividing the graduating class, which numbered 44, into small groups of seven members each, for the conferring of degrees, and having a short selection played by the orchestra while each group was retiring. The annual oration was delivered by the Rev. J. U. McCormick, and the valedictory by C. L. Morey, D.D.S., Texas. Twenty-four countries, states, and territories were represented in the graduating class. Matriculates, 118. Graduates, 44. Increase of matriculates over 1889 was 4. Per cent. of graduates to matriculates for 1889 was 37. For 1888, 46. Average for two years, 41.

The Chicago College of Dental Surgery held its seventh annual commencement at the Grand Opera House, Tuesday, March 26, 1889. The faculty address was made by Prof. Geo. H. Cushing, M.D., D.D.S., and the valedictory by Benjamin Eshelman, D.D.S., Iowa. Truman W. Brophy, M.D., D.D.S., Pres., conferred the degree of D.D.S. upon sixty-four graduates. An honorary degree was conferred upon James Atwood Swasey, Chicago. The number of matriculates was 154; graduates, 64; representing ten different countries, states and territories—29 being from Illinois.

Increase of matriculates over 1888, 18. Per cent. of graduates to matriculates for 1889, 41; for 1888, 34; average, 37.

The Indiana Dental College held its tenth annual commencement Wednesday, March 6th, at 8 P. M., at Association Hall, Indianapolis. Addresses were made by Dr. John Chambers and Prof. Coulter, of Wabash College. Degrees were conferred upon 17 aspirants. Matriculates for the year, 51; with nine different states represented in the graduating class.

Increase of matriculates over 1888, 21. Per cent. of graduates to matriculates for 1889, 33; for 1888, 54; average for two years, 43.

The Eighth annual commencement of the Kansas City Dental College was held Monday, March 11th, 1889, 8 o'clock P. M., Music Hall, Broadway, Kansas City, Mo.

Addresses on behalf of the faculty by C. B. Hewitt, D.D.S.

Conferring of degrees and award of prizes by E. W. Schauffler, President of Faculty.

Matriculates, 30; graduates, 11—representing five countries and States.

Increase of matriculates over 1888, 7. Per cent. of graduates to matriculates for 1889, 36. For 1888, 35. Average for two years, 35.

The Twenty-third annual commencement of the Missouri Dental College occurred at Memorial Hall, St. Louis, Missouri, March

14th, 1889. Prof. Henry Mudd, Dean, conferring the degree of Doctor of Dental Surgery upon eighteen aspirants for dental honors. The honorary degree was given Chas. R. E. Koch, Chicago, Ill. Number of matriculates fifty-four. The graduating class represented four States and Territories and two fareign countries.

Increase of matriculates over 1888, 19. Per cent. of graduates to matriculates for 1889, 38. For 1888, 37. Average for two years, 35.

The New York College of Dentistry held its twenty-third commencement at Chickering Hall, New York City, Monday evening, March 4, 1889. The valedictory address was given by Dr. John Charles Oberle, D.D.S., of New York.

Number of matriculates for session, 245; graduates, 69; representing twenty-six counties and States, with 19 foreigners.

Increase of matriculates over 1888, 34. Per cent. of graduates to matriculates for 1889, 25. For 1888, 3; average for two years, 29.

The Forty-third annual commencement of the Ohio College of Dental Surgery was held on Monday, March 4, 1889, at College Hall, Cincinnati. E. D. Warfield, Esq., delivered the annual address, and H. M. Patton, D.D.S., the valedictory for the class.

C. R. Taft, Vice-President of the Board of Trustees, confirmed degrees upon 65 applicants, representing 15 different States; 33, however, being from Ohio. The whole number of matriculates was 150—an increase of 36 over 1888. Per cent. of graduates to matriculates for 1889, 43; for 1888, 26. Average for two years, 36.

The Thirty-third annual commencement exercises of the Pennsylvania College of Dental Surgery took place at the Academy of Music, Philadelphia, March 1, 1889. An address was delivered by Prof. Henry Leffman, D.D.S. The degrees were conferred by President S. W. Gross, since deceased. Full number of matriculates for session, 178. The graduating class were attired in full-dress suits and numbered 91. Increase of matriculates over 1888, 18. Per cent. of graduates to matriculates for 1889, 51; for 1888, 36. Average for two years, 43. We are informed, however, that this number included four names that had been in attendance three years, so that the regular class numbered 87.

The Philadelphia Dental College held its Twenty-sixth annual commencement exercises at the Academy of Music on the evening of February 28, 1889. This institution has adopted caps and gowns, which made a very pretty effect. The annual address was delivered by Prof. S. B. Howell, M.D., D.D.S., and the valedictory by James G.

Whiting, D.D.S. The degrees were confirmed by ex-Governor Pollock, President of the Board of Trustees. Matriculates for the session, 226. Graduates, 98, representing 29 different countries and States, with 23 foreign, of whom 14 were from Canada. Increase of matriculates over 1888, 13. Per cent. of graduates to matriculates for 1889, 43 ; for 1888, 54. Average for two years, 49.

The Second annual commencement of the Dental Department of the Columbian University was held at Washington, D. C., March 21, 1889, being held in the Albaugh Opera House.

Prof. Henry C. Thompson, D.D.S., delivered the address to the class which numbered fourteen, and presented three members for graduation representing two different States. Increase of matriculates over 1888,—. Per cent. of graduates to matriculates for 1889, 21; for 1888, —. Average for two years, —.

The Dental Department of Howard University held its Second annual commencement in the Congregational Church, Washington, D. C., March 16, 1889. Robert Rayburn made the address to the class, and Hamilton Smith delivered the valedictory.

Matriculates for the session, 11. Graduates, 6, representing four different States. Decrease of matriculates since 1888, 9. Per cent. of graduates to matriculates for 1889, 55. For 1888, 40. Average for two years, 47.

The Seventh annual commencement exercises of the Dental Department of the University of Iowa were held at the Opera House, Iowa City, March 4, 1889. Hon. Albert Swaine gave the address to the students. The degree of D.D.S., was conferred upon twenty-one aspirants for dental honors by Chas H. Schaeffer, President of the University. Number of matriculates for session 79. The senior class representing six different States. Quorum of matriculates over 1888, 29 ; per cent. of graduates to matriculates for 1889, 26. For 1888, 40. Average for two years, 38.

The Seventh annual commencement exercises of the Department of Dental Surgery of the University of Maryland took place Wednesday, March 13, 1889, at the Lyceum Theatre, Baltimore, Md., Prof. F. J. S. Gorgar read the mandamus, Dr. J. B. Patrick gave the address to the class, and Hampton K. Smith, D.D.S., delivered the valedictory. Degrees were conferred by Hon. S. Teakle Wallis, LL.D., Provost of the University, upon thirty-nine members of the class, which numbered one hundred and twenty for the session.

The graduating class represented twenty different countries and States. Increase of matriculates over 1888, 11. Per cent. of graduates to matriculates for 1889, 32 ; for 1888, 47 ; average for two years, 39.

The Dental Department of the University of Tennessee held its Eleventh Annual Commencement in the Masonic Theatre, Nashville, Tenn., on Tuesday, February 26, 1889. The annual address to graduates was made by Prof. W. E. McCambell, A.M., D.D.S., and the valedictory by Rubert D. Crutcher, D.D.S. Matriculates for the session, 22. Graduates upon whom the title of D.D.S. was conferred numbered 13, representing four different states.

Decrease in matriculation since 1888, 6. Per cent. of graduates for 1889, 69; For 1888, 32. Average for two years, 50.

The Annual Commencement excercises of the Dental Department of the St. Louis College of Physicians and Surgeons were held at Memorial Hall, St. Louis, Mo., Friday, March 8, 1889. Degrees were conferred upon six graduates in a class of fourteen representing — different states. Decrease of matriculates since 1888, 4. Per cent. of graduates to matriculates for 1889, 44. For 1888, 39. Average for two years, 41.

The Second Annual Commencement exercises of the Dental Department of the Southern Medical College, Atlanta, Ga., were held in De Give's Opera House, Atlanta, Ga., Saturday, March 2d, 1889. Rev. Dr. Walker was the orator of the occasion. The Valedictory was delivered by A. R. McBoth, D.D.S.

Thomas S. Towell, M.D., President of the Board of Trustees, conferred the degrees upon seventeen members of the class, which numbered thirty-six for the session. The senior class represented seven different countries and states. Decrease of matriculates since 1888, 8. Per cent. of graduates to matriculates for 1889, 47; For 1888, 34. Average for two years, 40.

The Third Annual Commencement exercises of the School of Dentistry of Meharry Medical Department of Central Tennessee College were held in Masonic Hall, Nashville, Tenn., February 21, 1889. The Faculty address was rendered by R. F. Boya, M.D., D.D.S., and the valedictory by James R. Potter, A.B., D.D.S. Degrees were conferred by the President of the Faculty, J. Braden, D.D., upon six candidates, representing four different states. Whole number of matriculatas, 11. Decrease of matriculates over 1888, 1. Per cent. of graduates to matriculates for 1889, 54. For 1888, 16. Average for two years, 35.

The Department of Dentistry of Vanderbilt University held its Tenth Commencement at the Masonic Theater, Nashville, Tenn., February 20th, 1889. The faculty address was delivered by Prof. D. R. Stubblefield, D.D.S., and the valedictory by J. McClark, D.D.S. The degree of D.D.S. was conferred by L. C. Garland, L.L.D.,

Chancellor of the University. Candidates for gradution, 28 ; matriculates for the session, 95. The senior class represented fourteen diffrent states. Increase of matriculates over 1888, 18 ; Per cent. of graduates to matriculates for 1889, 29 ; for 1888, 42 ; average for two years, 35.

